# A Chitosan Induced 9-Lipoxygenase in *Adelostemma gracillimum* Seedlings

**DOI:** 10.3390/ijms13010540

**Published:** 2012-01-05

**Authors:** Jing Li, Pei-Ji Zhao, Chang-Le Ma, Ying Zeng

**Affiliations:** 1 State Key Laboratory of Phytochemistry and Plant Resources in West China, Kunming Institute of Botany, Chinese Academy of Sciences, Kunming 650204, China; E-Mails: lijing@mail.kib.ac.cn (J.L.); plenty@mail.kib.ac.cn (P.-J.Z.); 2 Graduate School of the Chinese Academy of Sciences, Beijing 100049, China; 3 Life Science College, Southwest Forestry University, Kunming 650224, China; E-Mail: machangle@sina.com

**Keywords:** *Adelostemma gracillimum*, lipoxygenase, chitosan, trihydroxyl octadecenoic acid, functional expression, *AgLOX1*

## Abstract

Oxylipins generated by the lipoxygenase (LOX) pathway play an important role in plant defense against biotic and abiotic stress. In chitosan-treated *Adelostemma gracillimum* seedlings, obvious accumulation of 9-LOX-derived oxylipins, namely 9,10,11-trihydroxy-12-octadecenoic acid, was detected. Using degenerate primers, a LOX-specific fragment putatively encoding LOX was obtained by RT-PCR, and a 2.9-kb full-length cDNA named *AgLOX1* was isolated by RACE from chitosan-induced *A. gracillimum* seedlings. Genomic Southern analysis implied that there was only one copy of *AgLOX1* in the *A. gracillimum* genome. *AgLOX1* was expressed in *Escherichia coli* and the recombinant protein was partially purified. The enzyme converted linoleic and linolenic acids almost exclusively to their 9-hydroperoxides. *AgLOX1* encoded a 9-lipoxygenase. Northern blot analysis indicated that chitosan-induced *AgLOX1* transcript accumulation peaked at 8 h after initiation of treatment, whereas trihydroxy derivatives accumulation was highest at 24 h after elicitation. Results showed that chitosan-induced *AgLOX1* encoded a 9-lipoxygenase potentially involved in the defense response through 9-LOX pathway leading to biosynthesis of antimicrobial compounds in *A. gracillimum* seedlings.

## 1. Introduction

Lipoxygenases (LOXs; EC 1.13.11.12) are nonheme iron-containing dioxygenases widely distributed in plants and animals [1]. LOX catalyzes the regio- and stereo-specific oxygenation of polyunsaturated fatty acids containing a *cis*,*cis*-1,4-pentadiene system to produce an unsaturated fatty acid hydroperoxide [2]. The lipoxygenase reaction involves a stereo selective hydrogen removal from a doubly allylic methylene and a subsequent stereo-specific insertion of molecular oxygen [3]. Linoleic and linolenic acids are common substrates of plant LOXs. Since linoleic acid only contains one doubly allylic methylene (C-11), only the hydrogen at this carbon atom can be removed. Consequently, only two positional isomers, namely 13- and 9-hydroperoxy-octadecadienoic acid, may be formed [3]. One method of plant LOX classification, namely, 9-LOX and 13-LOX, follows the positional specificity [4]. Another is based on the comparison of their primary structure [5]. Most plant LOXs show high overall sequence similarity (~70%) and their encoded enzymes lack a putative chloroplast transit peptide which is designated as type-1 LOX. Some LOXs carry an *N*-terminal transit peptide, which exhibit a moderate overall sequence similarity (~40%) and are classified as type-2 LOX [5].

Many LOX genes are present in plants and are often induced under specific conditions or during plant development [6]. LOX activity is also induced by treatment of cell cultures and plants with elicitors [7]. Hydroperoxidation products derived from LOX activity are mainly substrates for other enzymatic systems that transform the highly reactive molecules into a series of oxylipins via the so-called “LOX pathway” [8]. A total of seven different enzyme families compete for hydroperoxy derivatives as substrates in plants [5], where the produced oxylipins play a role in plant growth, development, senescence in response to biotic or abiotic stress [8].

In plants, 9- and 13-LOX pathways yield distinct oxylipins. For example, cyclic compounds analogous to jasmonates originate from the 13-LOX pathway. However, some fungitoxic compounds such as divinyl ether and hydroxylated fatty acids are derived mainly from the 9-LOX pathway [8]. In the last decade, 13-LOX-derived oxylipins such as jasmonoids have drawn the attention of many scientists and their biological roles have been intensively studied [9]. However, the products of the 9-LOX pathway have only recently gained attention and the importance of 9-LOX has been recognized mainly in Solanaceous plants. In pathogen-infected potato leaves, 9-LOX-derived 9,10,11-trihydroxy derivatives accumulate and exhibit antimicrobial activity, which are involved in resistance response by direct inhibition of pathogen growth [10]. LOX metabolites accumulation such as hydroxyl fatty acids can be used as makers of lipid peroxidation, which are concomitant with increased LOX activity [11].

*Adelostemma gracillimum*, which belongs to Asclepiadaceae, is a plant family close to Solanaceae. It is a traditional Tibet folk medicine plant distributed in the alpine area of northern Myanma and northwestern Yunnan Province of China. We isolated two trihydroxy octadecenoic acids in the calli of *A. gracillimum.* They are 9,12,13-trihydroxy-10-octadecenoic acid and 9,10,11-trihydroxy-12-octadecenoic acid by MS and NMR analysis, respectively. They have the same molecular formula and the same molecular weight (*m/z* 330), but the retention time is 7 min and 10 min, respectively, according to LC-ESI-MS detection [12]. In chitosan induced *A. gracillimum* seedlings, 9,10,11-trihydroxy-12-octadecenoic acid, which was considered to be derived from the oxygenation product of 9-LOX [13], accumulated with the induction time. It therefore seems that the 9-LOX pathway is likely involved in the biosynthesis of metabolites functioning in chemical defense in this plant. However, the research about LOX is not yet reported in Asclepiadaceae. To understand the role of the antimicrobial oxylipins in the chitosan induced LOX pathway, we characterize the LOX gene and investigate the oxylipins production.

## 2. Results and Discussion

### 2.1. Isolation of a Lipoxygenase cDNA from Seedlings of *Adelostemma gracillimum*

Seedlings induced by chitosan for eight hours were used for RNA and mRNA isolation. By using a homology based PCR cloning strategy and RACE-PCR, a full-length cDNA clone of 2949 bp was obtained and designated as *AgLOX1* (GenBank ID: DQ094169). The open reading frame (ORF) of *AgLOX1* was 2592 bp encoding a polypeptide of 863 amino acids with a calculated molecular mass of 98.0 kDa and a predicted isoelectric point of 5.94.

**Figure 1:**
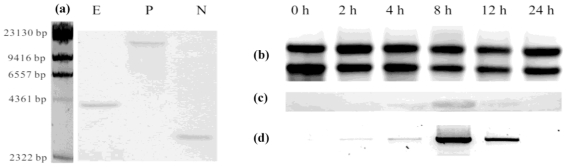
DNA and RNA gel blot and RT-PCR analysis of the *AgLOX1* gene. (**a**) Genomic Southern analysis of *AgLOX1*. Total genomic DNA (15 μg) was digested with either EcoR I, Pvu II, or Nco I, separated by electrophoresis on a 0.8% agarose gel, transferred to a positive Nylon membrane, and hybridized with a 484 bp coding region using λHindIII as a marker; (**b**) Ethidium bromide staining of total RNA; (**c**) Northern blot analysis of the *AgLOX1* transcripts in chitosan induced seedlings of different induction time (15 μg total RNA per lane), Chitosan-induced LOX transcript accumulation peaked at 8 h after initiation of treatment; (**d**) RT-PCR analysis of *AgLOX1* expressed in the chitosan induced seedlings of different induction time. The highest expression was also observed at 8 h after initiation of treatment. Equal amount of total RNA was used in the first-strand cDNA synthesis.

Southern blot analysis was carried out to examine the complexity of the *A. gracillimum* LOX gene family using the *AgLOX1* cDNA as probe. As documented in Figure 1a, under our experimental conditions, only one band was clearly visible, which indicated that there was only one copy of this gene in the *A. gracillimum* genome.

### 2.2. Comparison between AgLOX1 and Other Plant Lipoxygenase Genes

The deduced full-length amino acid sequence of *AgLOX1* was used to search for the translated sequences in GenBank. Database searches resulted in a number of plant lipoxygenase sequences. BLAST analysis revealed that, at the amino acid level, *A. gracillimum* LOX shared the highest degree of identity with the abscisic acid induced potato LOX (74%, GenBank ID: U60202) [14] and the elicitor-induced tobacco LOX (73%, GenBank ID: X84040) [15]. At the nucleic acid level, *AgLOX1* showed the highest identity with the toxin-induced LOX gene in tomato (79%, GenBank ID: AY008278) and the elicitor-induced LOX gene in tobacco (78%, GenBank ID: X84040) [15].

According to the deduced amino acids, the enzyme possessed the highly conserved C-terminal motif GIPNSVSI [1]. The sequence also contained some highly conserved regions responsible for the catalytic activity of the enzyme, including substrate binding domain KSAWRTDEEFAREMLA (positions 361–376) [1,16], oxygen binding domain IASALHAAVNFGQ (positions 710–722) [1], and five positions related to iron binding (His519, His524, His715, Asn719, and Ile863) [17]. The *A. gracillimum* LOX contained no transit peptide for chloroplast targeting according to sequence comparison, thus it could be classified as type-1 LOX [18]. The TV-motif, an indicator of a 9-LOX activity [19], was found in *AgLOX1* at the expected positions 581–582.

### 2.3. Functional Analysis of AgLOX1

**Figure 2:**
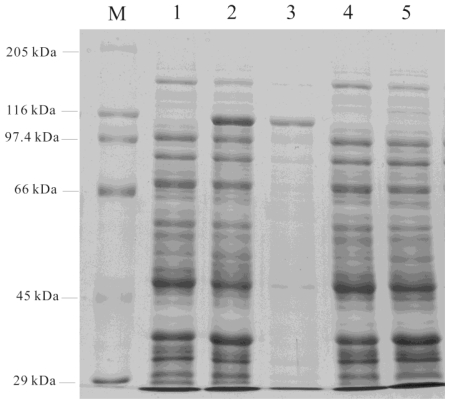
Purification of the His-tagged proteins analyzed by SDS-PAGE (8% polyacrylamide gel, Coomassie staining). Recombinant protein of 110.7-kD was expressed in a fusion pattern. Lane M: molecular mass Marker (Waltson, Shanghai). Lane 1: extracts from bacteria containing *Agl*/pET32a+ before IPTG induction. Lane 2: extracts from bacteria containing *Agl*/pET32a+ after IPTG induction for 20 h. Lane 3: purified recombinant protein. Lane 4: extracts from bacteria containing pET32a+ before IPTG induction. Lane 5: extracts from bacteria containing pET32a+ after IPTG induction for 20 h. Lane 4 and lane 5 were used as a negative control. There was no visible band present after IPTG induction.

Expression of *AgLOX1* in *E. coli* was achieved by a fusion expression using vector pET32a+. The ORF of *AgLOX1* was cloned into pET32a+, and the resulting plasmid *Agl*/pET32a+ was transferred to *E. coli* BL21 (DE3), using the original pET32a+ as a negative control. A protein band with an apparent molecular mass of about 116 kD was observed after induction with 1 mM IPTG for 20 h at 15 °C (Figure 2, lane 2). The analysis of the elution on SDS/PAGE led to the detection of one intense band with expected size of the recombinant protein following purification (Figure 2, lane 3). No corresponding band was detected in the negative control cells after IPTG induction (Figure 2, lane 5). The partially purified recombinant protein was dialysed against distilled water overnight and used in enzyme essays.

**Figure 3:**
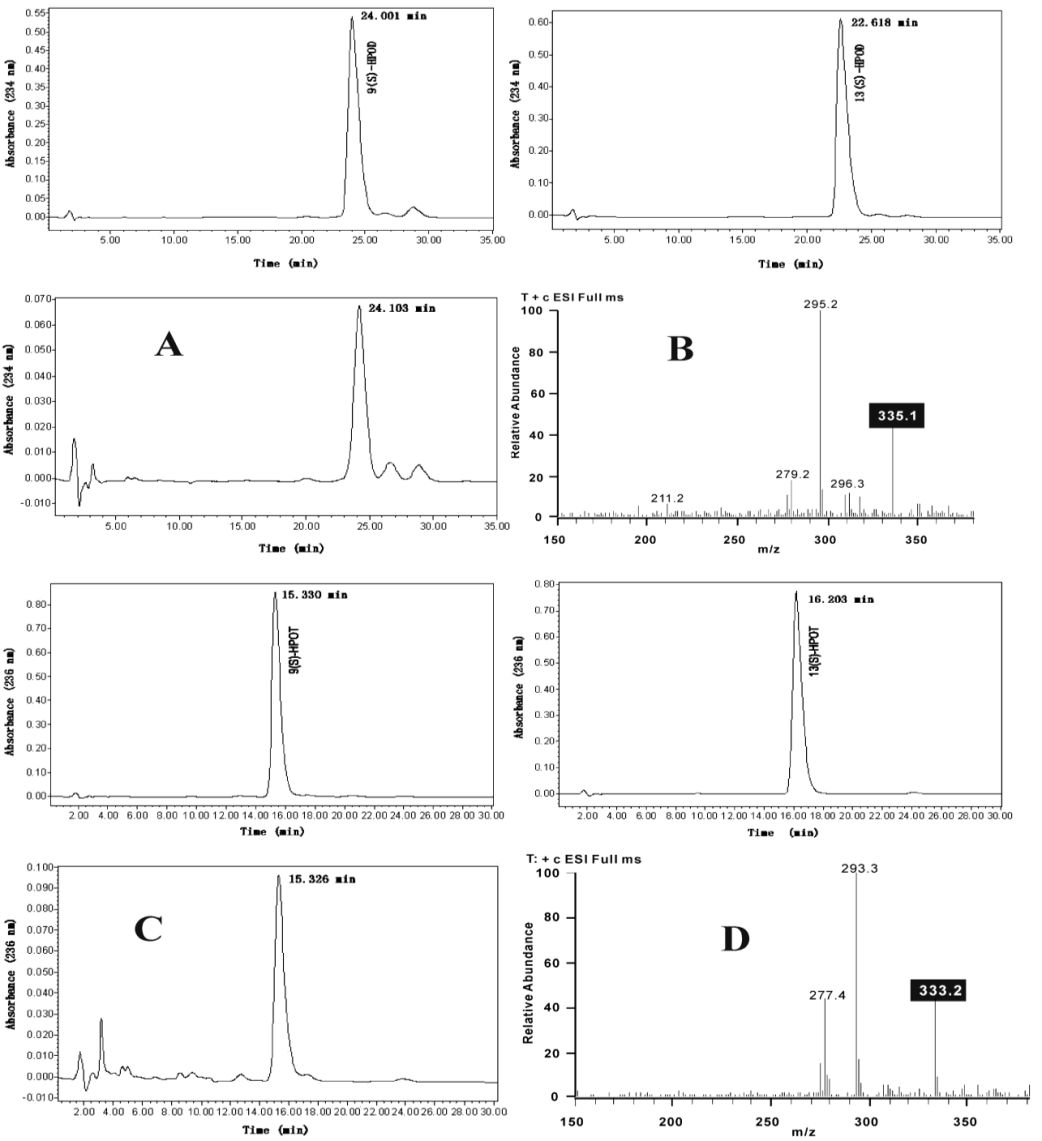
HPLC and LC-MS analysis of the products formed by recombinant LOX with linoleic acid (**A**, **B**) and linolenic acid (**C**, **D**) as substrates. Identification of the products were done by comparing their retention time with authentic standards 9(S)-HPOD, 13(S)-HPOD, 9(S)-HPOT and 13(S)-HPOT, and by analysis of the ion peaks in mass spectrum.

Using linoleic and linolenic acids as substrates, the partially purified lipoxygenase preferentially catalyzed the production of 9-hydroperoxide according to HPLC and MS results (Figure 3). As shown in Figure 3, using linoleic acid as substrate, the product eluted at 24 min (Figure 3A) with the same retention time as authentic 9(S)-hydroperoxy octadecadienoic acid (HPOD), and from the mass spectrum, an obvious ion peak at *m/z* 335 [M + Na^+^] was observed (Figure 3B). Using linolenic acid as substrate, the product eluted at 15 min (Figure 3C) with the same retention time as authentic 9(S)-hydroperoxy octadecatrienoic acid (HPOT), and from the mass spectrum, an obvious ion peak at *m/z* 333 [M + Na^+^] was observed (Figure 3D). Negative control extracts or heat-treated target protein did not show any LOX enzyme activity.

### 2.4. Expression Analysis of AgLOX1 in Seedling upon Chitosan Treatment

**Figure 4:**
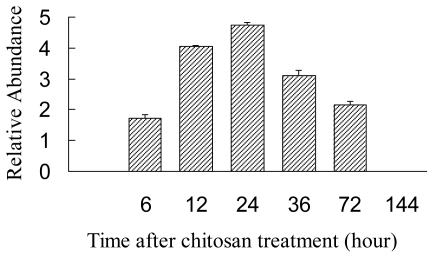
Time course of the relative abundance of 9,10,11-trihydroxy-12-octadecenoic acid in chitosan treated *A. gracillimum* seedlings. Trihydroxy derivatives accumulation was highest at 24 h after elicitation. Three parallel induction experiments were conducted and three samples of seedlings for each time were collected together for trihydroxyl octadecenoic acid extraction.

In chitosan-treated *A. gracillimum* seedlings, an obvious ion peak at *m/z* 353.5 was observed with the retention time from 9.4 to 10.4 min by LC-ESI-MS detection. Compound was identified to be (Z)-9,10,11-trihydroxy-12-octadecenoic acid based on spectroscopic data [12]. (Z)-9,10,11-trihydroxy-12-octadecenoic acid: colorless powder. ^1^H-NMR (400 MHz): 6.10 (1H, t, *J* = 9.2 Hz, H-12), 5.71 (1H, m, H-13), 5.18 (1H, t, *J* = 7.6 Hz, H-11), 4.44 (1H, m, H-9), 3.94 (1H, m, H-10), 2.49 (2H, t, *J* = 7.4 Hz, H-2), 2.22 (2H, m, H-14), 1.92–2.04 (2H, m, H-8), 1.49–1.58 (m, H-3/H-6/H-6A), 1.15– 1.35 (m, H-4/H-5/H-7/H-15/H-16/H-17), 1.77 (1H, m, H-6B), 0.79 (3H, t, *J* = 6.8 Hz, H-18). ^13^C-NMR (100 MHz): 176.0 (s, C-1), 132.6 (d, C-12), 132.3 (d, C-13), 76.9 (d, C-10), 71.3 (d, C-9), 69.3 (d, C-11), 34.8 (t, C-2/C-8), 31.7 (t, C-16), 29.6–30.1 (4t, C-4/C-5/C-7/C-15/C-14), 28.3 (t, C-14), 26.7 (t, C-6), 25.7 (t, C-3), 22.7 (t, C-17), 14.2 (q, C-18). ESI-MS: 353 [M + Na]^+^. Relative amount of 9,10,11-trihydroxide was determined by total MS chromatogram. The time course of the production of trihydroxy derivatives is shown in Figure 4. *A. gracillimum* seedlings accumulated maximal (Z)-9,10,11-trihydroxy-12-octadecenoic acid after being treated with 150 mg/L chitosan for 24 h. No obvious accumulation was detected in the water-treated seedlings.

Northern blot analysis using a specific *AgLOX1* RNA probe was conducted to explore the expression pattern of *AgLOX1* induced by chitosan. Figure 1c shows the *AgLOX1* mRNA levels in chitosan-induced *A. gracillimum* seedlings during the course of 24 h. The highest transitory accumulation occurred at 8 h following chitosan treatment. RT-PCR analysis (Figure 1d) showed a very similar pattern to Northern blot. Following chitosan treatment, the transcriptional level of *AgLOX1* accumulated with the induction time. Meanwhile, the final product of the 9-LOX pathway, 9,10,11-trihydroxy-12-octadecenoic acid accumulated with delay. However, no such phenomenon was observed in the water-treated *A. gracillimum* seedlings. This indicated the correlation between the *AgLOX1* expression and oxylipin production in chitosan-treated *A. gracillimum* seedlings.

## 3. Experimental Section

### 3.1. Plant Materials, Substrates and Reagents

The seeds of *A. gracillimum* were collected in Shangrila County, Yunnan Province, China, and stored at 4 °C. They were used for germination [20]. Standard linoleic acid and linolenic acid were purchased from Sigma. Restriction enzymes were obtained from New England Biolabs. T4-DNA ligase and pGEM-T Vector System I were purchased from Promega. All other biochemicals and reagents were purchased from Sigma or Amresco, unless otherwise noted. Primers were synthesized by Shanghai Sangon Co., and automated DNA sequencing was conducted at Shanghai GeneCore BioTechnologies Co.

Chitosan (Low molecular weight, Sigma) was prepared with the Komaraiah method [21]. The elicitor was applied to the final concentration of 150 mg/L. The seedlings of 14 days after inoculation were used for chitosan treatment. Intact plants were sprayed with 150 mg/L chitosan solution as described by Farmer and Ryan [22]. Control cultures were treated with distilled water. Three parallel induction experiments were conducted and three samples of seedlings for each time were collected together for trihydroxyl octadecenoic acid extraction.

### 3.2. Extraction and LC-ESI-MS Analysis of Trihydroxy Octadecenoic Acids

About 0.6 g of fresh seedlings (after chitosan-treatment for 0, 6, 12, 24, 36, 72, and 144 h) were extracted three times with acetone. The filtrate was evaporated to dryness under vacuum below 40 °C and the residue was dissolved in 1 mL MeOH. The samples were prepared for LC analysis after filtration through a 0.25-μm filter.

The samples were subjected to gradient elution on a reverse phase HPLC system (HPLC Waters 2695, C18 reversed-phase column 3.5 μm, 3.0 × 50 mm), where the mobile phase solution A is methanol and solution B is distilled water containing 1% formic acid. A multistep gradient was set up for trihydroxy octadecenoic acids estimation, with an initial injection volume of 10 μL and a flow rate 0.2 mL/min. ESI-MS analysis was carried out with electrospray ionization mass spectrometer (ESI-MS) on Thermo Finnigan LCQ Advantage instrument. The retention time and molecular mass were monitored under the following conditions: 63% aqueous methanol (distilled water containing 1% formic acid) (0–20 min) and methanol (20–25 min) and 63% aqueous methanol (distilled water containing 1% formic acid) (25–30 min). The high gas temperature (275 °C) and gas flow (50 psi) were applied to the LC-MS parameters. Scan ranges of 200–600 amu were used for positive ions. The MS parameters were optimized using 9,10,11-trihydroxy-12-octadecenoic acid (*m/z* 353 [M + Na]) as the reference standard compound.

Standard 9,10,11-trihydroxy-12-octadecenoic acid was isolated from the callus of *A. gracillimum*. The callus (57 g, biomass dry weight DW) were collected and extracted three times with 95% acetone under refluxing to afford 4.53 g of crude extract. The extract was subjected to column chromatography over reversed-phase C_18_ Si gel (80 g) eluted with methanol-water (1:1, 3:2, 7:3, 8:2, v/v), and further purified with column chromatography over Si gel and Sephadex LH-20 to give compound (5.5 mg).

### 3.3. cDNA Synthesis and RACE

Total RNA was isolated using the RNAex Reagent and RNAex Reagent Systems (Watson, Shanghai) and mRNA was extracted from the total RNA using an mRNA purification kit according to the manufacturer’s recommendation (Amersham Pharmacia).

**Scheme 1:**
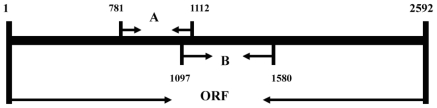
The open reading frame (ORF) of *AgLOX1*. (**A**) Original PCR fragment; (**B**) The probe used for Northern and Southern analyses.

First-strand cDNA was generated from the total RNA using the SuperScript™ First-Strand Synthesis System for RT-PCR (Invitrogen). This *ss-*cDNA was used as a template for PCR-based cloning. Two degenerate primers were designed based on the known plant lipoxygenases gene sequences, sense primer A: 5′-TAT YTN CCV ASY SAR CAN CC-3′ and antisense primer B: 5′-GCR AAY TCY TCR TCD GTI MWC CA-3′. The PCR reactions were carried out with a touch down program of 2 min denaturation at 95 °C, followed by 30 cycles of 30 s at 95 °C, 30 s from 55 °C to 40 °C, 1 min 30 s at 72 °C and terminated by 7 min extension at 72 °C. A 332 bp (from ^781^T to ^1112^C of ORF, Scheme 1) fragment of the expected size was cloned into pGEM-T vectors and six *E. coli* clones containing the original amplicon were sequenced. DNA sequences were analyzed using BLASTN and BLASTX programs of the NCBI [23]. The 3′-ends were amplified using specific primer designed according to the sequencing results (C: 5′-AGT GGT CCA AGT CCT TCT CCC-3′) and primer adaptor-oligo(dT)_16_.

The full-length cDNA template was synthesized from mRNA using SMART™ cDNA Library Construction Kit (Clontech) while the 5′-RACE was conducted according to the manufacturer’s recommendation. According to the sequence information of RACE, primers D: 5′-CGT ATC CAT AAC TGC AAA CAT ATC -3′ and E: 5′-GTG CAG TGT ATT CTG TTT AAA TAA AC-3′ were designed and used for the amplification of the full-length cDNA. The amplification of an approximate 3000 bp DNA fragment was cloned, sequenced and designated as *AgLOX1*. The complete *AgLOX1* cDNA sequence was submitted to the GenBank/EMBL database under an accession number of DQ094169.

### 3.4. Southern and Northern Analyses

Genomic DNA from the seedlings of *A. gracillimum* was isolated using a Plant DNA Maxi Kit (Watson, Shanghai). According to the cDNA sequence, a specific probe of 484 bp (from ^1097^C to ^1580^G of ORF, Scheme 1) was prepared using the PCR-DIG Probe Synthesis Kit (Roche), and was applied in genomic DNA hybridization analysis according to a standard procedure.

The total RNA of *A. gracillimum* seedlings was isolated using the RNAex Reagent and RNAex Reagent Systems (Watson, Shanghai). A specific fragment of 484 bp (from ^1097^C to ^1580^G of ORF, Scheme 1) used for RNA probe was prepared using the DIG RNA labeling Kit (Roche), and blots were hybridized for 16 h at 68 °C in DIG Easy Hyb buffer (Roche), washed and subjected to immunological detection (Roche, DIG Nucleic Acid Detection Kit).

### 3.5. Expression of cDNA in *E. coli*

In regards to the functional characterization of the encoded lipoxygenase, a full-length open reading frame of the cDNA was prepared from the defined clone *AgLOX*. Restriction sites of SalI and NotI for subcloning were introduced by PCR using primer combinations of Agl-SalI (5′-CCGTCGAC AA ATG CTG AAA CAA ATT C-3′) and Agl-NotI (5′-CAGCGGCCGC TTA AAT TGA CAC ACT G-3′). The amplified products were cloned and sequenced. Following the verification of the sequence, the SalI-NotI fragments were subcloned into the vector pET32a+ (Novagen) to yield the plasmid Agl/pET32a+. Plasmids were transformed into *E. coli* BL21 (DE3) for a fusion expression, using the original pET32a+ as negative control.

Following the cultivation of 20 h at 15 °C in the presence of 1 mM IPTG, the *E. coli* cells with Agl/pET32a+ were collected and suspended in 1 × binding buffer (0.5 M NaCl, 20 mM Tris-HCl, 5 mM imidazole, pH 7.9) and disrupted by sonication. The particulate was removed by centrifugation (6000 × g, 10 min), and the supernatant was used for the purification of the enzyme.

Purification of His-tagged proteins was performed according to the Ni-NTA His•Bind Superflow protocol (Novagen). Protein concentration was determined by the method of Brandford with BSA (Sigma) as a standard. All fractions were analyzed with SDS/PAGE on 8% polyacrylamide gel at 150 V for 1.5 h.

### 3.6. Enzyme Assays and Products Analysis

About 0.1 mg of the purified protein was added to 100 mM sodium phosphate buffer (pH 6.0) containing linoleic or linolenic acids (60 μM, dissolved in anhydrous ethanol which was diluted to be 1% at the final concentration), and the mixture (1 mL) was shaken vigorously for 1 h at 20 °C. The reaction was quenched by adding 1 mL of ethyl acetate and thoroughly mixed by vortexing, and centrifuged at 6000 g for 5 min and the ethyl acetate phase was collected. The extraction was repeated twice where the ethyl acetate evaporated under a stream of N_2_ and reconstituted in 1 mL anhydrous ethanol.

The reaction products were analyzed by RP-HPLC (Waters 996 HPLC system coupled to a photodiode array detector) using a symmetry C_18_ 5 μm column (Waters, 3.9 × 150 mm). A solvent system of CH_3_CN-H_2_O-CH_3_COOH (55:45:0.1, v/v/v) with a flow rate of 0.6 mL/min was used with an absorbance rate of 234 nm (linoleic acid as substrate) and 236 nm (linolenic acid as substrate) were recorded. Authentic standards of 9- and 13-hydroperoxides were purchased from Cayman Chemical (Ann Arbor, MI). Mass spectrums of the peak isolated from HPLC analysis were reanalyzed using LC-MS. Scan ranges of 150–500 amu were used for positive ions.

## 4. Conclusions

Plants are continually exposed to biotic and abiotic stresses and thus have formed various sophisticated defense mechanisms which involve very complicated cross-talking modulating systems and include activation of a series of genes and metabolic pathways [10]. Chitosan and other fungal elicitors are potent elicitors of plant defense responses and can induce the *de novo* synthesis of antimicrobial phytoalexins through the octadecanoid pathway [24]. Products of LOX pathway, namely oxylipins, are involved in the response, both as antimicrobial compounds and as signal molecules that lead to the activation of specific defense genes [25].

In our research, a full length lipoxygenase cDNA, designated as *AgLOX1*, was isolated in an Asclepiadaceae plant for the first time. The cDNA was expressed in *E. coli* and the recombinant protein was partially purified using His band resins. The enzyme activities showed that *AgLOX1* encoded a 9-lipoxygenase. In chitosan treated *A. gracillimum* seedlings, *AgLOX1* transcription and 9-LOX pathway oxylipins, 9,10,11-trihydroxy-12-octadecenoic acid, accumulated with induction time. Trihydroxy-octadecenoic acid was synthesized from linoleic acid-derived epoxy alcohols via epoxy alcohol synthase (EAS) and epoxy alcohol hydrolase [26], which has proven antifungal activities [27]. We concluded that chitosan-induced *AgLOX1* encoded a 9-lipoxygenase, potentially involved in the defense response through the 9-LOX pathway, leading to antimicrobial compounds biosynthesis in *A. gracillimum* seedlings.
